# Cross-cultural adaption and psychometric validation of Importance of Good Death-Indonesian version for patients with advanced cancer

**DOI:** 10.1016/j.apjon.2025.100798

**Published:** 2025-10-08

**Authors:** Wahyu Dewi Sulistyarini, Sheng-Yu Fan, Mei-Chih Huang, Christantie Effendy, Dongjuan Xu, Ting-Jyun Chen, Chi-Yin Kao

**Affiliations:** a Department of Nursing, Institute of Health Technology and Science Wiyata Husada Samarinda, Indonesia; b Institute of Gerontology, College of Medicine, National Cheng Kung University, Tainan, Taiwan; c National Tainan Junior College of Nursing, Tainan, Taiwan; d Department of Nursing, College of Medicine, National Cheng Kung University, Tainan, Taiwan; e Department of Medical Surgical Nursing, Faculty of Medicine, Public Health and Nursing, Universitas Gadjah Mada, Yogyakarta, Indonesia; f School of Nursing, Purdue University, West Lafayette, IN, USA

**Keywords:** Cancer, Death and dying, Palliative care, Psychometric testing

## Abstract

**Objective:**

To adapt and test the psychometric properties of the Importance of Good Death Questionnaire for Indonesian patients with advanced cancer.

**Methods:**

The original instrument underwent a rigorous process of forward and backward translation, complemented by an expert panel review to ensure cultural and conceptual equivalence and to evaluate content validity. A total of 447 patients with advanced cancer were recruited via convenience sampling from two government hospitals in Solo and Samarinda, Indonesia, between September 2022 and February 2023. The sample was then divided into two groups. Sample 1 (*n* ​= ​265) was used for exploratory factor analysis to establish the initial factor structure of the Indonesian version, and Sample 2 (*n* ​= ​182) was used for confirmatory factor analysis to validate construct validity. No significant differences in demographics or clinical variables were observed between the two samples.

**Results:**

Following the expert panel review, the instrument underwent exploratory factor analysis and confirmatory factor analysis, resulting in a final Indonesian version containing 24 items across five factors: comfort, relationship closure, preparation for death, support from others, and life meaning. The five-factor model accounted for 59.1% of the total variance. Specifically, comfort explained 40% of the variance, relationship closure explained 9.2%, preparation for death explained 5%, support from others explained 2.8%, and life meaning explained 2.1%. Internal consistency was excellent, with an overall Cronbach's alpha of 0.91 and factor-specific alpha values ranging from 0.73 to 0.93.

**Conclusions:**

The study results indicate that the Importance of Good Death-Indonesian version has good psychometric properties and is a valid and reliable instrument to assess the importance of good death in an Indonesian advanced cancer population. In nursing practice, the instrument can guide culturally responsive end-of-life care by identifying patients’ priorities across five domains. This enables nurses to develop individualized care plans, address pain and symptom needs, facilitate meaningful family interactions, support spiritual or practical preparation for death, and inform palliative care decision-making.

## Teaser

Talking about death is never easy; yet understanding what makes a “good death” is essential for improving care at the end of life. This study adapted and tested the Importance of Good Death questionnaire for use with Indonesian patients with advanced cancer. Through surveys with 447 patients, the Indonesian version identified five key areas people value most: comfort, relationship closure, preparation for death, support from others, and life meaning. The tool showed strong reliability and cultural relevance, reflecting Indonesia's spiritual traditions and family-centered values. For nurses and other health professionals, this tool offers practical guidance to tailor care, including managing pain, supporting family connections, and addressing spiritual needs. Therefore, patients can face the end of life with dignity and peace.

**Lay summary**

Talking about death is never easy, yet understanding what makes a “good death” is essential for improving care at the end of life. This study adapted and tested the Importance of Good Death questionnaire for use with Indonesian patients with advanced cancer. Through surveys with 447 patients, the Indonesian version identified five key areas people value most: comfort, relationship closure, preparation for death, support from others, and life meaning. The tool showed strong reliability and cultural relevance, reflecting Indonesia’s spiritual traditions and family-centered values. For nurses and other health professionals, this tool offers practical guidance to tailor care, such as managing pain, supporting family connections, and addressing spiritual needs. Therefore, patients can face the end of life with dignity and peace.

## Introduction

In the face of a global rise in cancer incidence and mortality, particularly in developing countries, the need for effective palliative care is increasingly critical.^1^ Understanding the elements of a good death, which include physical, psychological, and spiritual comfort, is essential for delivering high-quality end-of-life care for cancer patients.^2^ In Indonesia, cultural and religious beliefs are deeply embedded in the dying process. As a predominantly Muslim country, spiritual readiness, dying in peace with God, and the presence of family are regarded as essential components of a good death.^3^ Furthermore, Indonesia's collectivist culture places a strong emphasis on family involvement in medical decisions and caregiving, which influences patient values, preferences, and expectations near the end of life.^4^ Despite these culturally distinct perspectives, there is a lack of validated instruments that can accurately capture what constitutes a good death from the viewpoint of Indonesian patients. Existing tools often reflect individualistic notions of autonomy and personal control, which may not fully resonate with Indonesian cultural norms. Therefore, adaptation of the Importance of Good Death (IGD) questionnaire^5^ to the Indonesian context offers the advantage of providing a culturally attuned assessment tool. This study addresses the gap by adapting and validating the IGD questionnaire for Indonesian patients with advanced cancer.

In 2022, the global cancer burden reached an estimated 20.0 million new cases and 9.7 million deaths. Asia accounted for nearly half of this burden, with 9,826,539 new cases and 5,464,451 deaths in the same year,^6^ the region is projected to maintain this disproportionate share, contributing approximately 49.2% of global incidence and 56.1% of mortality by 2025.^7^ In Indonesia specifically, there were 408,661 new cancer cases and 242,988 deaths in 2022, making it one of the highest burdens in Southeast Asia. As the cancer mortality rate gradually rises, the need for more effective palliative care for dying patients and a more compassionate dying process becomes increasingly important.^1^ As cancer progresses and becomes unresponsive to treatment, patients may feel lost and afraid in the face of the reality of approaching death.^8^ Understanding a patient's personal experience regarding death and dying can help alleviate their fear of death and lead to the development of approaches to handle it. Health professionals offering palliative care may want to ask patients to describe what they hope for at the end of life and how to achieve a good death in order to identify their care goals.^9^

The core elements of a good death include physical, psychological, and spiritual comfort, support from family and health professionals, and contributions to others.^10^ Researchers also highlight that the place of death, life completion, and dying with dignity are important components of a good death.^10^^,^^11^ However, these elements are culturally mediated. Several studies have explored the concept of a good death in East Asian countries with different findings. Japanese patients consider physical comfort and autonomy significantly more important than religion, preparation, and dying at home, while Taiwanese patients prefer life completion and being free from tubes and machines. Korean patients mentioned that remaining cognitively intact is significantly important.^12^ A study conducted in Thailand identified preference domains of a good death in three areas: not being a burden to others, preparation for death, and physical and psychological comfort.^13^ Therefore, a good death may be influenced by personal values, beliefs, social connections, religion, and cultural contexts.^14^

Indonesia is the most populous Muslim country, where people believe that illness is a test from God, an undeniable reality. People draw comfort from prayer, worship, and spiritual surrender (tawakkal) as a way to gain strength and peace.^15^ Family caregiving is integral to Indonesian culture, with extended family and community actively involved in providing support.^15^ The importance of accepting one's destiny (nrimo ing pandum) is a core Javanese value that shapes attitudes toward suffering and dying. Within this cultural framework, spiritual and familial elements often outweigh individual preferences or biomedical control over death.^16^

A systematic review identified the IGD as a psychometrically strong tool commonly used to assess a good death.^17^ The IGD was developed by Miyashita et al.^5^ and was applied to cancer populations and health professionals with 57 items across 18 factors, covering diverse dimensions including physical and psychological comfort, dying in a favorite place, good relationship with medical staff, maintaining hope and pleasure, not being a burden to others, good relationship with family, physical and cognitive control, environmental comfort, being respected as individual, life completion, natural death, preparation for death, role accomplishment and contributing to others, unawareness of death, fighting against cancer, pride and beauty, control over the future, and religious and spiritual comfort (Appendix A).^5^ The overall Cronbach's alpha ranged from 0.61 to 0.88.^18^ Participants rated the importance level of components of a good death on a seven-point Likert scale, ranging from 1 (absolutely unnecessary) to 7 (absolutely necessary). The higher mean score indicated a greater importance of the good death factor.^5^ The IGD has been successfully adapted in East Asia contexts such as China and South Korea, where cultural congruence with Confucian values has been emphasized. However, no validated instrument has been developed for use in the Indonesian context.^19^^,^^20^

Indonesia presents a unique blend of Islamic beliefs, Javanese values, and strong familial caregiving traditions. These cultural and spiritual elements resonate with many core domains of the IGD, especially those related to spiritual readiness, dignity, and family support. Therefore, adapting the IGD offered the advantage of building on an already robust and multidimensional framework while tailoring it to Indonesian cultural realities. A culturally adapted IGD enables nurses to better assess and respond to patients' end-of-life preferences, ultimately supporting more holistic, respectful, and person-centered nursing care. Hence, the current study aimed to culturally adapt and validate the IGD to develop the IGD-Indonesian version (IGD-I) for patients with advanced cancer. The IGD-I could serve as a practical tool to guide nursing practice by helping nurses identify patient values, inform individualized care planning, and facilitate culturally sensitive communication, particularly important in Indonesia, where spiritual and familial considerations are integral to end-of-life care.

## Methods

This study followed the Guidelines for Reporting Reliability and Agreement Studies (GRRAS) to enhance methodological transparency and reporting quality.^21^ It was conducted in two phases: 1) instrument translation and cultural adaptation, including the translation process and assessment of content validity, and 2) psychometric testing, which involved construct validity through exploratory factor analysis (EFA) and confirmatory factor analysis (CFA) and reliability assessment via internal consistency. An independent expert review panel was established to provide comments and advice for each phase, ensuring that the IGD-I was comprehensible and culturally appropriate for the Indonesian context while preserving the original instrument's measurement properties.^22^ The expert review panel consisted of seven members: one nursing professor, four academics specializing in palliative care, and two clinical research scientists.

### Instrument translation and adaptation

The first phase involved the translation process, following the standard Beaton translation procedure to ensure a culture-adapted version.^23^ The original IGD with 57 items across 18 factors^5^ was first translated from English into Bahasa Indonesian using a forward and backward translation approach. Two academic nursing professionals, proficient in Indonesian and English, independently translated the original instrument into Bahasa Indonesia. After reconciliation through discussion, a single forward-translated version was produced.

Next, two additional bilingual experts, unfamiliar with the original instrument, independently back-translated the reconciled version into English. Two backward translations were then reviewed and synthesized into a single version. Discrepancies between the forward and back translations were identified and resolved through consensus discussions among the translators and the expert review panel. This process ensured that the translated items retained their original meaning while reflecting cultural nuances.

Subsequently, four additional experts, including nursing academics and clinical nurses, were invited to assess the content validity of the draft Indonesian version. Content validity was measured by the content validity index (CVI), including the item level (I-CVI) and the scale level (S-CVI) values, with acceptable CVI values greater than 0.78.^24^ Four items (original items 44, 50, 52, and 54 in Appendix A) were deleted due to cultural misalignment, and several items were revised according to the experts’ suggestions. Finally, the translated version of IGD-I with 53 items was completed with good content validity.

### Psychometric evaluation: exploratory and confirmatory factor analysis

#### Participants

A convenience sampling method was used to recruit participants. Patients with advanced cancer were invited from two government hospitals in Solo, Central Java, and Samarinda, East Kalimantan. Data collection was conducted between September 2022 and February 2023. Eligible participants met the following inclusion criteria included: 1) being diagnosed with advanced cancer; 2) being able to communicate in Bahasa Indonesian; 3) being able to concentrate at least for 20 minutes to complete the questionnaire; 4) aged 18 years or older; and 5) without a diagnosed mental illness which was reviewed by patients’ medical records to confirm the absence of mental illness or cognitive impairment as assessed by health professionals. For participants who could not read or write, a trained research assistant read the questionnaire and recorded their responses, ensuring that participants could ask questions or clarify items at any time.

The required sample size was determined based on the rule of thumb of at least five participants per item on the instrument.^25^ Given that the translated version of IGD-I consisted of 53 items, a minimum of 265 participants was required for EFA. Participants from Solo, Central Java (Sample 1; *n* ​= ​265), were used for the EFA, after which the number of items was further reduced. Participants from Samarinda, East Kalimantan (Sample 2; *n* ​= ​182), were then used for the CFA. In total, 447 participants were recruited to support both EFA and CFA, ensuring a robust psychometric evaluation. This division allowed for two independent samples to be used for two analytical procedures, aligning with best practices in psychometric testing.^26^

#### Measures

The study questionnaire included two parts: 1) demographics and clinical information, and 2) the translated version of IGD-I. Patient demographics included age, gender, marital status, religion, and household income, while clinical information included the cancer diagnosis, time since diagnosis, and performance status.^27^^,^^28^ The Eastern Cooperative Oncology Group (ECOG) Performance Scale was used to assess the patient's performance status with scores ranging from 0 to 5 (0 ​= ​fully active and able to carry all pre-disease performance without restrictions, 5 ​= ​death).^29^ The translated version of IGD-I contained 53 items that we aimed to develop and validate in this study.^5^ We used a seven-point Likert scale to rate the importance of each item (1: absolutely unnecessary to 7: absolutely necessary). The scale does not contain any reverse-coded items. Average scores are calculated for the overall scale and each subscale, ranging from 1 to 7. Higher scores indicate a greater perceived importance of a good death.

#### Data collection

Data collection was carried out by four trained research assistants, all of whom held a bachelor's degree in nursing and had prior experience in research data collection. Prior to data collection, the research team conducted a structured briefing session that covered the study objectives, data collection procedures, inclusion/exclusion criteria, and instrument usage. A simulation exercise was also conducted to ensure consistency and standardization across all assistants.

These steps were implemented to enhance the reliability and quality of data collection. Participants’ demographics and clinical information were collected by the research staff, and if participants could read and write, the main questionnaire (the translated version of the IGD-I) with an envelope was provided to participants to complete. Once the questionnaire was completed, participants returned it to the research team.

#### Data analysis

SPSS version 22 software for Windows was used to perform descriptive statistics for demographic and clinical variables and to conduct the EFA. The corrected item–total correlation was applied to assess whether individual items effectively represented the underlying psychological construct. A value lower than 0.3 was set as an indicator that an item should be omitted.^30^ Based on these criteria, seven items were removed (original items 15, 34, 43, 49, 51, 53, and 55 in Appendix A), resulting in the refined version of IGD-I with 46 items for the EFA.

The Kaiser-Meyer-Olkin (KMO) measure of sampling adequacy was 0.900, which exceeded the recommended value of 0.8, and Bartlett's test of Sphericity reached statistical significance (*P* ​< ​0.001), supporting the suitability of the data for factor analysis. Because the items in the refined version of IGD-I were expected to be intercorrelated, principal axis factoring with Promax rotation was used. EFA was conducted using data from Sample 1 (*n* ​= ​265) to explore the underlying structure. Items were removed based on two criteria: 1) the factor loading less than 0.32, and 2) substantial cross-loadings with other factors. Only one item was removed at a time, followed by a repeated factor analysis to reassess item performance.^26^ The number of factors was determined using two criteria: 1) an eigenvalue ≥ 1 and 2) inspection of the screen plot.

CFA was then conducted using LISREL version 8.80 with data from Sample 2 (*n* ​= ​182) to validate the factor structure identified through EFA. Model fit was evaluated based on the following fit indices: Standardized Root Mean Square Residual (SRMR), Root Mean Square Error of Approximation (RMSEA), Comparative Fit Index (CFI), and Incremental Fit Index (IFI). Higher CFI and IFI values (> 0.90) and lower RMSEA (< 0.08) and SRMR (< 0.08) values indicate a good fit.^31^ The final version of the IGD-I was confirmed at this stage. Cronbach's alpha was used to evaluate the internal consistency of the IGD-I questionnaire and its subscales.

## Results

### Demographics

A total of 447 patients participated in this study: 265 in Sample 1 and 182 in Sample 2. Comparing the participant characteristics between these two samples, no significant differences in demographics or clinical variables were found (Table 1). Overall, participants ranged in age from 20 to 95 years (Mean ​= ​54.7, standard deviation [SD] ​= ​12.3) with the majority being female (*n* ​= ​322; 72.0%) and married (*n* ​= ​414; 92.6%). More than 94% of the participants (*n* ​= ​421) were Muslim, and nearly 82% of the participants (*n* ​= ​366) had household incomes of less than USD 125 per month. Breast cancer (*n* ​= ​119; 26.6%) and cervical cancer (*n* ​= ​113; 25.3%) were the top two cancer diagnoses. Nearly 69% (*n* ​= ​307) had been diagnosed with cancer within the year prior. Nearly 78% of participants (*n* ​= ​347) were able to perform self-care by themselves (ECOG < 2). All participants received medical treatments during the data collection.

**Table 1:** Demographic and clinical characteristics in Samples 1 and 2.

Variables	Sample 1 (*n* ​= ​265)		Sample 2 (*n* ​= ​182)		Total (*N* ​= ​447)	
	*n*	%	*n*	%	*n*	%
**Sex**						
Male	80	30.2	45	24.7	125	28.0
Female	185	69.8	137	75.3	322	72.0
**Marital status**						
Single	8	3.0	4	2.2	12	2.7
Married	243	91.7	171	94.0	414	92.6
Widowed	6	2.3	2	1.1	8	1.8
Divorce	8	3.0	5	2.7	13	2.9
**Religion**						
Muslim	246	92.8	175	96.2	421	94.2
Christian	17	6.4	3	1.6	20	4.5
Catholic	1	0.4	4	2.2	5	1.1
Hindu	1	0.4	0	0	1	0.2
**House hold income per month**						
< ​125 USD	203	76.6	163	89.6	366	81.9
125 – 250 USD	48	18.1	16	8.8	64	14.3
> 250 USD	14	5.3	3	1.6	17	3.8
**Diagnosis**						
Breast cancer	71	26.8	48	26.4	119	26.6
Cervical cancer	62	23.4	51	28.0	113	25.3
Lung cancer	21	7.9	16	8.8	37	8.3
Ovarian cancer	10	3.8	12	6.6	22	4.9
Rectal cancer	17	6.4	11	6.0	28	6.3
Nasopharyngeal cancer	32	12.1	17	9.3	49	11.0
Other	52	19.6	27	14.8	79	17.7
**Time since diagnosis**						
Less than 6 months ago	87	32.8	66	36.3	153	34.2
6 month – 1 year ago	92	34.7	62	34.1	154	34.5
1 year – 2 years ago	46	17.4	26	14.3	72	16.1
More than 2 years ago	40	15.1	28	15.4	68	15.2
**ECOG performance status**						
0: Able to carry on all pre-disease performance	102	38.5	69	37.9	171	38.3
1: Restricted in term of physically strenuous activity	98	37.0	78	42.9	176	39.4
2: Capable of self-care but unable to carry out work activities	49	18.5	23	12.6	72	16.1
3: Only limited self-care	16	6.0	12	6.6	28	6.3

ECOG, Eastern Cooperative Oncology Group.

### EFA

The EFA initially extracted 10 factors with eigenvalues > 1, which explained 63.6% of the variance; however, the scree plot revealed a clear break after the fifth component, supporting the retention of a five-factor solution. During the iterative factor refinement process, items with factor loadings less than 0.32 or substantial cross-loadings were systematically removed one at a time, followed by repeated analyses. A total of 22 items (original items 2, 5, 6, 8, 10, 13, 17, 18, 19, 20, 21, 23, 24, 25, 27, 28, 33, 41, 45, 47, 48, and 57 in Appendix A) were removed based on these criteria. All deletions were reviewed and approved by the expert review panel to ensure conceptual integrity and cultural appropriateness of the remaining items. This process resulted in a final structure comprising 24 items across five factors, explaining 59.1% of the total variance. Most items showed factor loadings greater than 0.40, except for one item (having family support), which was retained based on theoretical relevance. The first factor of seven items represented comfort (40% of variance); the second factor of four items related to relationship closure (9.2%); the third factor of four items reflected preparation for death (5%); the fourth factor of four items referred to support from others (2.8%); and the fifth factor of five items represented life meaning (2.1%) (Table 2).

**Table 2:** Factor loading for the EFA and reliability in Samples 1 and 2.

Items	Factor loadings					Reliability of subscale in Sample 1/2
	Comfort	Relationship Closure	Preparation for Death	Support from Others	Life Meaning	
**Comfort**						0.83/0.79
1. Being able to stay at one's favorite place	0.751					
2. Having some pleasure in daily life	0.664					
3. Living in calm circumstances	0.703					
4. Being free from pain & physical distress	0.687					
5. Not being a burden to family members	0.612					
6. Being independent in daily activities	0.556					
7. Being respected for one's values	0.499					
**Relationship closure**						0.90/0.93
8. Seeing people whom one wants to see		0.893				
9. Feeling thankful to people		0.680				
10. Being reconciled with people		0.676				
11. Saying good bye to loved ones		0.642				
**Preparation for death**						0.86/0.84
12. Family has no regrets for one's death			0.911			
13. Having no regrets			0.850			
14. Feeling that one's life is complete			0.728			
15. Being prepared for dying			0.663			
**Support from others**						0.84/0.82
16. Receiving consistent care from the same physician and nurse				0.912		
17. Having a nurse with whom one feels comfortable				0.855		
18. Trusting physician				0.808		
19. Having family support				0.392		
**Life meaning**						0.76/0.73
20. Feeling that one can contribute to others					0.588	
21. Fighting against the disease until one's last moment					0.530	
22. Having faith					0.561	
23. Living in hope					0.512	
24. Feeling that one's life is worth living					0.442	

### CFA

**Fig. 1:**
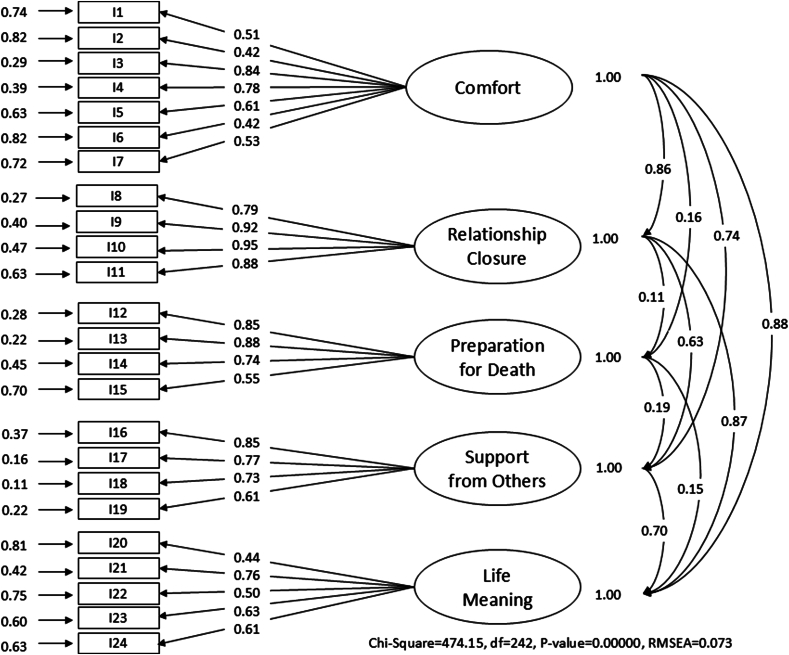
Confirmatory factor analysis for the Indonesian version of the Importance of Good Death Questionnaire. RMSEA, Root Mean Square Error of Approximation.

A five-factor model consisting of 24 items, as identified through EFA, was tested using CFA in Sample 2. The model demonstrated a good model fit to the data, with fit indices as follows: CFI ​= ​0.95, IFI ​= ​0.95, RMSEA ​= ​0.073, and SRMR ​= ​0.065 (Fig. 1).

### Internal consistency

The final version of the IGD-I demonstrated good internal reliability across both samples. The overall Cronbach's alpha was 0.91. In Sample 1, subscale reliabilities were as follows: comfort (*α* ​= ​0.83), relationship closure (*α* ​= ​0.90), preparation for death (*α* ​= ​0.86), support from others (*α* ​= ​0.84), and life meaning (*α* ​= ​0.76). In Sample 2, reliability estimates remained consistent: comfort (*α* ​= ​0.79), relationship closure (*α* ​= ​0.93), preparation for death (*α* ​= ​0.84), support from others (*α* ​= ​0.82), and life meaning (*α* ​= ​0.73). These results indicate acceptable to excellent internal consistency across all five factors (Table 2).

## Discussion

### Main findings

This study represents the effort to culturally adapt and psychometrically validate the IGD questionnaire for use among Indonesian patients with advanced cancer. By doing so, it contributes to both the theoretical understanding and practical application of what constitutes a good death in a culturally specific context. While the concept of a good death has been studied globally, it is inherently influenced by local cultural, religious, and societal values.^32^^,^^33^ The adaptation process of the IGD into the Indonesian version involved both empirical and contextual considerations, ensuring its relevance to the cultural setting and its applicability in nursing practice to support patient-centered end-of-life care.

Of the original 57 items, four were removed due to a lack of alignment with Indonesian cultural expectations, and 29 additional items were excluded due to conceptual irrelevance or psychometric weakness, resulting in a concise 24-item instrument with strong internal consistency across five factors. This reduction was not merely statistical but culturally meaningful, reflecting Indonesian beliefs that prioritize family harmony, spiritual preparedness, and acceptance of life's course over individual control and autonomy in the dying process.^34^ Unlike in more individualistic cultures where personal autonomy is emphasized, Indonesian end-of-life perspectives center around communal decision-making, religious submission (tawakkal), and accepting fate (nrimo ing pandum).^34^^,^^35^ These values are embedded in the final version of the IGD-I.

A five-factor structure emerged through both EFA and CFA, supporting the construct validity of the IGD-I. The internal consistency of the overall instrument was strong (Cronbach's alpha ​= ​0.91), with reliability coefficients for the five factors, comfort, relationship closure, preparation for death, support from others, and life meaning, ranging from 0.73 to 0.93. These results affirm the psychometric robustness of the IGD-I and also reflect Indonesian patients' perceptions of what matters most at the end of life. Having a good death is an important individualized experience at the end of life for cancer patients.^33^ Understanding what constitutes a good death is a critical point in end-of-life care,^5^ assisting nurse as health professional in delivering appropriate care and developing individualized nursing care plans for cancer patients.^36^

In Javanese cultural philosophy about life and death, it is stated that life is a loan and must be returned to its owner when the time comes. It is called as *oncating sukma saka raga* in Javanese language. Another important expression, *surud ing kasedan jati, titis ing pati* reflects the hope that when facing death. It means when people face death, they hope to find peace and a good and appropriate death atmosphere so they will return to God well.^37^ This concept is in accordance with the goals of palliative care which hope that the patients will be able to achieve a good death in their dying process.

In this study, the *comfort* factor comprised staying at a favorite place, maintaining pleasure, living in calm circumstances, being free from pain and physical distress, not being a burden to family members, and being independent and respected. Several studies have pointed out comfort as the most important aspect of good death.^10^^,^^32^^,^^33^^,^^36^^,^^38^ A previous study showed that being free from pain was reported by 81% of participants, including patients, health professionals, and family members, as an item describing a good death.^33^ Other literature supported this finding and mentioned that comfort has various concepts, including the point of physical, psychosocial, spiritual, and environmental aspects such as pain and symptom management, creating a calm and peaceful environment.^39^

*Relationship closures* factor refers to seeing people whom one wants to see, feeling thankful toward others, being reconciled with people, and saying goodbye to loved ones. The literature also supports this point. When asked what gives people's lives purpose, most cite their close, personal ties with someone they love, and fulfilling and long-lasting relationships are essential to happiness and overall well-being.^40^ Existing literature discussed the impact of relationship completion on easing the latter days of life. It also highlighted the turmoil experienced when those relationships were unresolved or in chaos. These five sentiments allow relationships to be completed once they are communicated: "I love you," "Thank you," "Forgive me," "I forgive you," and "Goodbye".^40^

*Preparation for death* is also an important part of achieving a good death. Dying has been described as the final phase in the life process for a person. In the present study, this factor includes four items: family members have no regrets about one's death, one has no regrets, feeling that one's life is complete, and being prepared for dying. Preparation for death is defined as preparation for the death of a loved one, with patients being resolved over important issues such as financial matters, last will, and funeral arrangements after death.^8^ Literature also points out that preparation for death includes emotional and psychological readiness to face death, which can significantly alleviate anxiety and provide peace for patients and their families.^33^^,^^40^^,^^41^ Studies have shown that individuals who engage in preparatory actions, such as discussing their end-of-life wishes with loved ones, nurses or other health care professionals, experience greater control and completeness in their final days.^33^

The factor *support* *from others* refers to family support and health professional support, aligning with the previous discussion about a peaceful death that integrates personal, medical, and social elements to achieve a peaceful death.^42^ The literature indicates that relationships with health professionals and others are one of the core themes of a good death.^33^ Meanwhile, family support includes the constant presence of family members, accompanying patients through the dying process, and strengthening relationships.^38^ In Indonesian culture, particularly for sick persons, their relatives are highly involved in direct care. When a family member is ill, the rest of the family members gather to give love and empathy and pray for the sick person.^43^ Therefore, caring for family members is part of Indonesian culture. It may be the way individuals appreciate life and inheritance.^44^ Family and community support play a significant role in providing care to sick and dying patients in Indonesia.^45^

The factor of *life meaning* includes contributions to others, fighting the disease, faith, and hope, and a sense that one's life is worth living. Religion and spirituality are important means by which individuals find meaning, purpose, and value in their lives.^46^ Faith, as part of spirituality, plays an important role in helping people cope with suffering.^47^^,^^48^ A previous study in Indonesia showed that 91% of advanced cancer patients have spiritual needs,^43^ with another study indicating that advanced cancer patients seek spiritual well-being to deal with their illness.^49^ Faith teaches people to live in hope and prevents them from living in despair.^50^ Hope contributes and influences people's search for meaning after they are diagnosed with cancer.^51^

The study eliminated four factors from the original IGD, including pride and beauty, natural death, unawareness of death, and control over the future. This is based on religion and cultural beliefs in Indonesia. Previous Indonesian studies showed that body image and general appearance were not priority problems for cancer patients.^52^ In terms of natural death, unawareness of death and control over the future were contraindicated with Indonesian culture and beliefs. This is because Muslim people have strong beliefs in accepting illness, believing that illness, suffering and dying are a test from God and nobody can ignore this reality.^15^ In Islamic belief, spiritual medicine from their faith empowers them to face their situation; hence, people tend to believe that God will help them, and they gain more strength by surrendering to God, trying hard, and not despairing or called *tawakal*.^53^ It indicates that patients believe that all treatments can help them fight cancer until the last moment.

Muslims and Javanese believe the disease is an expiation of sins during life and reduces the sins that are borne when you die.^54^^,^^55^ Even though patients understand their poor disease prognosis, they choose not to think of death as a consequence of illness, preferring to maintain hope for a cure and discover an alternative treatment for their diseases. As a result, people focus on symptom control and close relationships with family and address their faith to fight the disease.^56^ Some people, however, believe sick persons should react passively and obediently without asking questions as the best way to get health care. In Javanese cultures, the concept of *Nrimo ing pandum* reflects an attitude of fully accepting what life gives, including becoming sick and having to deal with the pain throughout his life. This attitude is thought to help reduce depression and become strong in facing trials of pain and not giving up.^16^

### Implications for nursing practice and research

The IGD-I also offers significant clinical utility. It can enable health professionals to identify and prioritize patients' values and needs, thereby, informing the development of individualized care plans, and supporting culturally appropriate communication. In palliative care settings, the IGD-I can guide discussions about end-of-life preferences and ensure that care interventions align with patients’ spiritual beliefs and social-cultural contexts. This is particularly important in Indonesia, where culturally adapted assessment tools remain limited and both patients and nurses have voiced the need for more culturally responsive communication instruments.

Beyond its assessment function, the IGD-I provides practical implications for nursing interventions in Indonesia by translating patients' domain-specific priorities into tailored end-of-life care. For example, when patients place high importance on comfort, nurses can focus on effective pain and symptom management, support independence in daily activities, and help reduce feelings of being a burden to family. Strong emphasis on relationship closure may guide nurses to facilitate family visits, encourage reconciliation, and create opportunities for expressing gratitude or saying goodbye. A high priority on preparation for death highlights the need for supporting spiritual counseling, life review, and practical arrangements that reduce regret for both patients and families. Similarly, when support from others is valued, nurses can strengthen continuity of care, foster trustful nurse–patient relationships, and mobilize family or community involvement. Finally, prioritizing life meaning calls for integrating dignity-conserving practices, spiritual support, and opportunities for legacy building into individualized care plans. Beyond bedside care, aggregated IGD-I results can inform staff training by highlighting common cultural preferences and areas needing enhanced sensitivity, thereby strengthening palliative care services. Ultimately, the tool empowers nurses to deliver holistic, culturally congruent care that aligns with patients’ values and enhances satisfaction for both patients and families.

### Limitations

Several limitations of this study should be acknowledged. First, the study population was comprised of patients with advanced cancer, rather than terminally ill patients who are often too weak and cognitively impaired to complete a 20-min questionnaire. Considering the disease trajectory, advanced cancer patients may have greater awareness of their prognosis and thus represent an appropriate population for exploring perceptions of a good death. However, this may limit the generalizability of the findings to terminally ill populations. Future validation of the IGD-I with terminal patients is recommended. Secondly, although efforts were made to recruit participants from both Java and Kalimantan, the majority were Muslim. This limits the generalizability of the findings across Indonesia's diverse religious and ethnic populations. Future studies should aim to validate the IGD-I among non-Muslim populations, individuals residing in other regions or islands, and patients with terminal illness other than cancer. Comparative studies examining cultural and religious differences in perceptions of a good death would enhance the instrument's national applicability and support its use as a standard for culturally competent end-of-life care. Third, one limitation of this study is the uneven distribution of explained variance across the five factors, with the first factor (comfort) accounting for a large proportion (40%) and the remaining factors contributing relatively small percentages (ranging from 9.2% to 2.1%). This pattern is partly attributable to the use of Promax rotation, which assumes intercorrelations among factors. In such cases, variance is shared across factors, and the unique variance attributed to each subsequent factor may appear reduced. This does not necessarily indicate that these constructs are weak, but rather reflects the interrelated nature of the dimensions underlying the concept of a good death. These findings suggest the need for future qualitative and longitudinal research to further refine and validate the culturally meaningful components of a good death in the Indonesian context.

## Conclusions

In conclusion, this study is the first to culturally adapt and validate the Importance of Good Death questionnaire for use in Indonesia, resulting in a psychometrically sound 24-item instrument (IGD-I) including five factors: comfort, support from others, relationship closures, preparation for death, and life meaning. These factors reflect Indonesian cultural and religious values that emphasize communal decision-making, spiritual readiness, and family harmony over individual autonomy. The IGD-I not only offers theoretical insight into culturally grounded perceptions of a good death but also provides a practical tool for nurses and other health professionals to assess patients' end-of-life preferences, guide sensitive communication, and deliver more personalized and culturally sensitive palliative care. Future research should extend validation across Indonesia's diverse populations to promote its broader clinical application.

## CRediT authorship contribution statement

**Wahyu Dewi Sulistyarini:** Conceptualization, Methodology, Investigation, Project Administration, Formal analysis, Data Curation, Writing – Original Draft. **Sheng-Yu Fan:** Conceptualization, Methodology, Formal analysis, Validation, Data Curation, Writing – Review & Editing. **Mei-Chih Huang:** Validation, Resources, Writing – Review & Editing. **Christantie Effendy:** Validation, Resources, Writing – Review & Editing. **Dongjuan Xu:** Data Curation, Validation, Writing – Review & Editing. **Ting-Jyun Chen:** Validation, Software, Writing – Review & Editing. **Chi-Yin Kao:** Conceptualization, Methodology, Validation, Formal analysis, Data Curation, Writing – Review & Editing, Supervision, and Funding acquisition. All authors have read and approved the final manuscript.

## Ethics statements

The study was approved by the Institutional Review Boards of the Moewardi Hospital in Central Java (Approval No. 710/1X/HREC) and RSUD Abdoel Wahab Sjahranie Hospital in East Samarinda (Approval No. 492/KEPK-AWS/III) and was conducted in accordance with the 1964 Helsinki Declaration and its later amendments or comparable ethical standards. All participants provided written informed consent.

## Data availability statement

The data that support the findings of this study are available on request from the corresponding author, C.-Y. Kao. The data are not publicly available due to ethical restrictions.

## Declaration of generative AI and AI-assisted technologies in the writing process

During the preparation of this work, the authors used ChatGPT to improve language and readability. It is important to note that the assistance provided by ChatGPT was limited to language editing, while all the original content, ideas, data analysis, results, and discussion were solely generated by the authors. After using this tool, the authors reviewed and edited the content as needed and take full responsibility for the content of the publication.

## Funding

This research was supported in part by Higher Education Sprout Project, 10.13039/100010002Ministry of Education to the Headquarters of University Advancement at 10.13039/501100007750National Cheng Kung University (NCKU). The funders had no role in considering the study design or in the collection, analysis, interpretation of data, writing of the report, or decision to submit the article for publication.

## Declaration of competing interest

All authors declare no conflicts of interest.
