# Supplementary material for: Cross-cultural adaption and psychometric validation of Importance of Good Death-Indonesian version for patients with advanced cancer
Source: Asia Pac J Oncol Nurs. 2025 Oct 8;12:100798. doi: 10.1016/j.apjon.2025.100798 (PMC12605861; doi:10.1016/j.apjon.2025.100798)
Supplement: Multimedia component 1 [file mmc1.docx]

**Appendix A.**

Comparison of items and factors between the original Importance of Good Death (IGD) questionnaire and the Indonesian version (IGD-I)

| **Original item (factor) in original IGD** | **Reason for deletion**  **(if applicable)** | **Final item in IGD-I** | **Final factor in IGD-I** |
| --- | --- | --- | --- |
| 1. Being free from pain & physical distress (Physical and psychological comfort) |  | 1. Being free from pain & physical distress | Comfort |
| 1. Being calm (Physical and psychological comfort) | Factor loading < 0.32 |  |  |
| 1. Being able to stay at one’s favorite place (Dying in a favorite place) |  | 1. Being able to stay at one’s favorite place | Comfort |
| 1. Trusting physician (Good relationship with medical staff) |  | 1. Trusting physician | Support from Others |
| 1. Discussing one’s treatment with one’s physician (Good relationship with medical staff) | Cross-loadings |  |  |
| 1. Having people who listen to me (Good relationship with medical staff) | Factor loading < 0.32 |  |  |
| 1. Receiving consistent care from the same physician and nurse (Good relationship with medical staff) |  | 1. Receiving consistent care from the same physician and nurse | Support from Others |
| 1. Having a physician or nurse with whom one can discuss fears of death (Good relationship with medical staff) | Cross-loadings |  |  |
| 1. Having a nurse with whom one feels comfortable (Good relationship with medical staff) |  | 1. Having a nurse with whom one feels comfortable | Support from Others |
| 1. Living positively (Maintaining hope and pleasure) | Cross-loadings |  |  |
| 1. Having some pleasure in daily life (Maintaining hope and pleasure) |  | 1. Having some pleasure in daily life | Comfort |
| 1. Living in hope (Maintaining hope and pleasure) |  | 1. Living in hope | Life Meaning |
| 1. Not making trouble for others (Not being a burden to others) | Cross-loadings |  |  |
| 1. Not being a burden to family members (Not being a burden to others) |  | 1. Not being a burden to family members | Comfort |
| 1. Having no financial worries (Not being a burden to others) | Item-total correlation < 0.3 |  |  |
| 1. Having family support (Good relationship with family) |  | 1. Having family support | Support from Others |
| 1. Having family by whom one can express one’s feeling (Good relationship with family) | Cross-loadings |  |  |
| 1. Spending enough time with one’s family (Good relationship with family) | Cross-loadings |  |  |
| 1. Family is prepared for one’s death (Good relationship with family) | Factor loading < 0.32 |  |  |
| 1. Believing that one’s family will do well after one’s death (Good relationship with family) | Factor loading < 0.32 |  |  |
| 1. Having family by one’s side when one is going to die (Good relationship with family) | Factor loading < 0.32 |  |  |
| 1. Being independent in daily activities (Physical and cognitive control) |  | 1. Being independent in daily activities | Comfort |
| 1. Being able to eat (Physical and cognitive control) | Cross-loadings |  |  |
| 1. Being mentally clear (Physical and cognitive control) | Factor loading < 0.32 |  |  |
| 1. Living like being at home (Environmental comfort) | Cross-loadings |  |  |
| 1. Living in calm circumstances (Environmental comfort) |  | 1. Living in calm circumstances | Comfort |
| 1. Not being treated as an object or a child (Being respected as an invidual) | Factor loading < 0.32 |  |  |
| 1. Being free from trivial routines (Being respected as an individual) | Factor loading < 0.32 |  |  |
| 1. Being respected for one’s values (Being respected as an individual) |  | 1. Being respected for one’s values | Comfort |
| 1. Feeling that one’s life was completed (Life completion) |  | 1. Feeling that one’s life is complete | Preparation for Death |
| 1. Family has no regrets for one’s death (Life completion) |  | 1. Family has no regrets for one’s death | Preparation for Death |
| 1. Having no regrets (Life completion) |  | 1. Having no regrets | Preparation for Death |
| 1. Not being connected to medical instruments or tubes (Natural death) | Factor loading < 0.32 |  |  |
| 1. Dying a natural death (Natural death) | Item-total correlation < 0.3 |  |  |
| 1. Being prepared for dying (Preparation for death) |  | 1. Being prepared for dying | Preparation for Death |
| 1. Seeing people whom one wants to see (Preparation for death) |  | 1. Seeing people whom one wants to see | Relationship Closure |
| 1. Saying good bye to dear people (Preparation for death) |  | 1. Saying good bye to loved ones | Relationship Closure |
| 1. Feeling thankful to people (Preparation for death) |  | 1. Feeling thankful to people | Relationship Closure |
| 1. Being reconciled with people (Preparation for death) |  | 1. Being reconciled with people | Relationship Closure |
| 1. Feeling that one can contribute to others (Role accomplishment and contributing to others) |  | 1. Feeling that one can contribute to others | Life Meaning |
| 1. Maintaining one’s role in family or occupational circumstances (Role accomplishment and contributing to others) | Factor loading < 0.32 |  |  |
| 1. Feeling that one’s life is worth living (Role accomplishment and contributing to others) |  | 1. Feeling that one’s life is worth living | Life Meaning |
| 1. Not being informed of bad news (Unawareness of death) | Item-total correlation < 0.3 |  |  |
| 1. Dying without awareness that one is dying (Unawareness of death) | Cultural misalignment |  |  |
| 1. Living as usual without thinking about death (Unawareness of death) | Factor loading < 0.32 |  |  |
| 1. Fighting against disease until one’s last moment (Fighting against cancer) |  | 1. Fighting against the disease until one’s last moment | Life Meaning |
| 1. Believing that one used all available treatments (Fighting against cancer) | Cross-loadings |  |  |
| 1. Living as long as possible (Fighting against cancer) | Factor loading < 0.32 |  |  |
| 1. Not receiving pity from others (Pride and beauty) | Item-total correlation < 0.3 |  |  |
| 1. Not having a change in ones appearance (Pride and beauty) | Cultural misalignment |  |  |
| 1. Not exposing one’ sphysical and mental weakness to anyone else (Pride and beauty) | Item-total correlation < 0.3 |  |  |
| 1. Controlling time of death, like euthanasia (Control over the future) | Cultural misalignment |  |  |
| 1. Having planned arrangements for one’s grave, funeral and last will (Control over the future) | Item-total correlation < 0.3 |  |  |
| 1. Knowing how long one will live (Control over the future) | Cultural misalignment |  |  |
| 1. Knowing what to expect about one’s condition the future (Control over the future) | Item-total correlation < 0.3 |  |  |
| 1. Having faith (Religious and spiritual comfort) |  | 1. Having faith | Life Meaning |
| 1. Feeling that one is protected by higher power beyond oneself (Religious and spiritual comfort) | Factor loading < 0.32 |  |  |
